# Reinforcement Learning-Based Energy Management of Smart Home with Rooftop Solar Photovoltaic System, Energy Storage System, and Home Appliances

**DOI:** 10.3390/s19183937

**Published:** 2019-09-12

**Authors:** Sangyoon Lee, Dae-Hyun Choi

**Affiliations:** School of Electrical and Electronics Engineering, Chung-Ang University, 84 Heukseok-ro, Dongjak-gu, Seoul 156-756, Korea; sangyoon1207@naver.com

**Keywords:** home energy management system, reinforcement learning, artificial neural network, smart home, consumer comfort, smart grid

## Abstract

This paper presents a data-driven approach that leverages reinforcement learning to manage the optimal energy consumption of a smart home with a rooftop solar photovoltaic system, energy storage system, and smart home appliances. Compared to existing model-based optimization methods for home energy management systems, the novelty of the proposed approach is as follows: (1) a model-free Q-learning method is applied to energy consumption scheduling for an individual controllable home appliance (air conditioner or washing machine), as well as the energy storage system charging and discharging, and (2) the prediction of the indoor temperature using an artificial neural network assists the proposed Q-learning algorithm in learning the relationship between the indoor temperature and energy consumption of the air conditioner accurately. The proposed Q-learning home energy management algorithm, integrated with the artificial neural network model, reduces the consumer electricity bill within the preferred comfort level (such as the indoor temperature) and the appliance operation characteristics. The simulations illustrate a single home with a solar photovoltaic system, an air conditioner, a washing machine, and an energy storage system with the time-of-use pricing. The results show that the relative electricity bill reduction of the proposed algorithm over the existing optimization approach is 14%.

## 1. Introduction

With the advent of the Internet of Things (IoT) technology, smart sensors, and advanced communication and control methods in electric energy systems, increasing amounts of electric energy-related data are being produced and utilized for the reliable and efficient operation of electric energy system. Machine learning (ML) is a core technology for handling such big data effectively, and various ML-based applications are currently under development for the prediction of solar photovoltaic (PV) generation, load forecasting, energy control and cost optimization, peak load management, and the design of dynamic energy pricing using various ML models, such as the artificial neural network (ANN), support vector machine, and deep learning [1]. This study attempts to provide a novel ML-based framework with which to conduct optimal energy management of residential homes.

Owing to the increasing home energy consumption [2] along with emerging smart grid technologies in the residential sector, such as distributed energy resources (DERs) (for example, rooftop PV systems and residential energy storage systems (ESSs)), advanced metering infrastructure with smart meters, and demand response programs, home energy management is becoming increasingly crucial for residential consumers to reduce their electricity bills and maintain the efficiency of their home appliances. Furthermore, as additional smart home appliances using the IoT technology, including air conditioners, washers, and refrigerators, are being deployed to offer more advanced services to consumers, the development of more intelligent systems, namely home energy management systems (HEMSs), is becoming necessary for consumers to control their home appliances efficiently and economically [3].

The HEMS is a key solution for residential energy management in future smart grids. The HEMS has two major functions: (1) monitoring the real-time energy use of consumers using smart meters and smart plugs, and (2) scheduling the optimal energy consumption of home appliances to reduce consumer electricity bills in their comfortable and preferred environments. A traditional core technology in HEMSs is the optimization method for economic load reduction and load shifting. In general, the HEMS algorithm is formulated as an optimization problem, in which the objective functions (consumer electricity bill and discomfort cost) are minimized while satisfying the equality and inequality constraints (such as the operations of appliances and DERs, and the consumer comfort settings).

**Figure 1:**
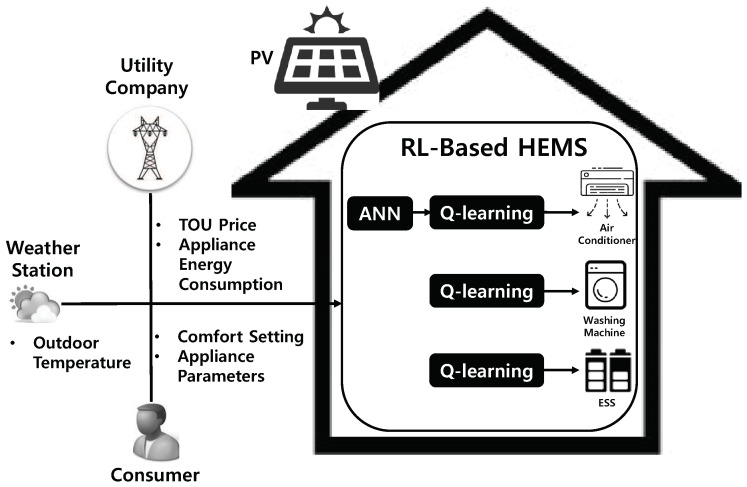
Illustration of the proposed home energy management system (HEMS) framework.

Compared to the existing model-based HEMS optimization approaches, we propose a HEMS algorithm using a model-free reinforcement learning (RL). Figure 1 presents the conceptual system model for the proposed RL-based HEMS, along with the classification of data associated with the utility company, weather station, and consumer. The main contributions of this paper are summarized as follows:

- We present an RL-based HEMS model that manages the optimal energy consumption of a smart home with a rooftop PV system, ESS, and smart home appliances. In the HEMS model, the Q-learning method is applied to the energy consumption scheduling of different home appliances (air conditioner, washing machine, and ESS), whereby the agent of each appliance determines the optimal policy independently to reduce its own electric cost within the consumer comfort level and the appliance operation characteristics. Furthermore, we propose an ANN model to learn the relationship between the indoor temperature and energy consumption of the air conditioner more accurately, which is integrated into the Q-learning module to achieve improved performance of the air conditioner agent.
- The simulation results confirm that the proposed RL method with the ANN can successfully reduce both the consumer electricity bill and dissatisfaction cost (for example, the indoor temperature and operating time interval of the washing machine within the consumer comfort settings). Moreover, we compare the performance of the proposed RL-based HEMS algorithm to that of the conventional mixed-integer linear programming (MILP)-based HEMS algorithm, and verify that the proposed approach can achieve greater energy savings than the conventional approach under various penalty parameter settings in the reward function of the appliance agent.

The remainder of this paper is organized as follows. Section 2 provides a literature review for our proposed method. Section 3 defines the various types of smart home appliances and introduces the conventional optimization formulation for home energy management. Section 4 presents the formulation of the proposed RL-based HEMS algorithm using the Q-learning and ANN methods. The simulation results for the proposed HEMS algorithm are provided in Section 5 along with discussion for the future applicability of the proposed algorithm in Section 6, and conclusions are provided in Section 7.

## 2. Related Research

Over the past decade, various studies have been conducted on the HEMS optimization formulation in different types of optimization models and performance assessment [4,5,6,7,8,9,10,11,12,13,14,15,16]. These approaches include the scheduling of different types of home appliances along with electric vehicles using linear programming (LP) [4,5], load scheduling considering the consumer comfort level using mixed integer nonlinear programming (MINLP) [6], convex programming based on relaxed MINLP using an $L_{1}$ regularization technique [7], load scheduling for a single consumer or multiple consumers using MILP [8,9,10], LP-based joint optimization of energy supplies and electric loads through three-stage scheduling (prediction, supply control, and demand control) [11], the natural aggregation algorithm (NAA)-based HEMS method consisting of forecasting, day-ahead scheduling, and actual operation [12], robust optimization for the scheduling of home appliances to resolve the uncertainty of consumer behavior [13] and the outdoor temperature and consumer comfort levels [14], and distributed HEMS architectures consisting of a local and global HEMS [15,16]. More recently, using real-time pricing, a HEMS optimization method that considers the operational dependency of various types of home appliances and consumer lifestyle requirements was proposed in [17]. Previous work on HEMS algorithms, including different types of optimization models, is summarized effectively in [18]. In addition, a broader literature review on the energy and comfort management of the residential, commercial and industrial buildings was conducted in [19].

In recent years, compared to the aforementioned model-based HEMS optimization approaches, data-driven approaches using ML methods have gained popularity owing to their more efficient residential energy management because the existing model-based approach is limited to deterministic decision-making under an uncertain environment and approximated energy system models, thereby leading to undesired energy consumption scheduling. In [20], an operation method for smart thermostats was presented, in which the consumer preference could be learned using a Bayesian inference method. Moreover, based on the learned consumer preference, the optimal temperature setting schedule for smart thermostats could be determined in a stochastic expected value model. A novel pooling-based deep recurrent neural network (RNN) method for household load forecasting was proposed to improve the accuracy significantly in [21]. Compared to the traditional deep RNN technique, a key method in [21] was learning spatial information among consumers and allowing for additional learning layers prior to the occurrence of overfitting. The numerical examples demonstrated that the proposed approach outperformed existing ML methods, such as the auto-regressive integrated moving average, support vector regression, and traditional deep RNN. In [22], a hybrid HEMS algorithm that integrated the ML methods into a traditional HEMS optimization problem was developed, in which the energy consumption of the heating, ventilation, and air conditioning (HVAC) was scheduled based on neural network-and regression-based learning methods. Furthermore, for a reliable wind energy management, a hybrid wind speed multi-step forecasting model was developed using an ANN method combined with the wavelet packet and complete ensemble empirical mode decomposition techniques in [23]. In [24], the Elman neural network that is optimized by the multi-objective salp swarm algorithm was used to enhance both the forecasting accuracy and stability of air quality early-warning system that improves air quality and human health. A hybrid electricity price forecasting method was presented in multi-step ahead framework, which consists of fast ensemble empirical mode decomposition, variational mode decomposition, and back propagation neural network in [25]. In [26], the ANN model was used to develop a tool that investigates the relationship among heating energy use, indoor temperatures, and the heating energy demand in the residential buildings with different occupant behaviors.

More recently, reinforcement learning (RL), also known as the model-free control approach, has received attention as a promising ML method for electric energy management. A pioneering work in RL-based energy management is Google DeepMind, which was developed using the RL method and has been proved to decrease the electricity bill by cooling the data center by approximately 40%. Deep RL (DRL) (that is, the combination of RL and ANN) was applied to the control of HVAC in a building to reduce the energy cost while maintaining a comfortable consumer level in terms of the indoor temperature [27], as well as both the indoor temperature and air quality [28]. Several papers have reported on building energy management with DERs using Q-learning, in which the ESS was controlled to achieve energy savings in a single [29] building and a community with multiple buildings [30]. In [31], multi-agent RL was presented to manage the home energy consumption. Each agent corresponded to various home appliances types with non-shiftable, shiftable, and controllable loads, and the energy consumption of each appliance was optimized through the Q-learning process, along with the real-time price prediction using the ANN. Recently, a novel Q-learning method using action dependent dual heuristic programming was proposed to solve the infinite-time domain linear quadratic tracker without requiring the information of the system matrices in [32]. In [33], Q-learning-based multi-agent framework was developed where all agents communicate with each other and synchronize with the leader agent, consequently achieving the optimal consensus solution for all agents in real time.

Although extensive research has been conducted on residential energy management using the RL method, to the best of the authors’ knowledge, no study has proposed an energy consumption scheduling algorithm yet considering the operation of various home appliances, including the ESS, and the consumer comfort level simultaneously. Previous studies have been limited to the energy consumption scheduling problem for controlling only the HVAC [27,28] or only the ESS [29,30]. Similarly to our work, the study of [31] developed a Q-learning-based HEMS algorithm that scheduled the energy consumption of different home appliances with shiftable and controllable loads. However, no control for the ESS charging and discharging was considered.

## 3. System Model for HEMS

### 3.1. Preliminary

In this study, we consider the situation in which automatic energy management for a single household is carried out by the HEMS, which schedules and controls the following types of household appliances under the time-of-use (TOU) tariff:

- Controllable appliance ($A^{c}$): A controllable appliance is an appliance of which the operation is scheduled and controlled by the HEMS. The operation characteristics categorize controllable appliances into reducible appliances ($A_{r}^{c}$) and shiftable appliances ($A_{s}^{c}$). An example of a reducible appliance is an air conditioner, known as a thermostatically controllable load, in which the energy consumption can be curtailed to reduce the electricity bill. However, under the TOU pricing scheme, the energy consumption of a shiftable appliance can be shifted from one time slot to another to minimize the total electricity cost. A shiftable appliance has two load types: (1) a non-interruptible load ($A_{s}^{c,NI}$), and (2) an interruptible load ($A_{s}^{c,I}$). The operation of shiftable appliances with non-interruptible loads must not be stopped by the HEMS control during the appliance task period. For example, a washing machine must perform a washing cycle prior to drying. A shiftable appliance with an interruptible load may be interrupted at any time. For example, the HEMS must terminate the discharging process and initiate the charging process of the ESS instantly when the PV power generation is greater than the load demand.
- Uncontrollable appliance ($A^{uc}$): An uncontrollable appliance, such as a TV, PC, or lighting, cannot be scheduled and operated by the HEMS. Therefore, $A^{uc}$ maintains the fixed energy consumption scheduling.

### 3.2. Conventional HEMS Optimization Formulation

A general HEMS algorithm that determines the optimal operating schedule of household appliances and DERs is formulated as an MILP optimization problem, consisting of the objective function and constraints as follows:

#### 3.2.1. Objective Function

The objective function (1) for the HEMS optimization problem consists of two parts, each of which includes different decision variables ($E_{t}^{\mathrm{net}},T_{t}^{\mathrm{in}}$):

(1) $$\min_{E_{t}^{\mathrm{net}},T_{t}^{\mathrm{in}}}\underset{J_{1}\left(E_{t}^{\mathrm{net}}\right)}{\underset{︸}{\sum_{t\in T}\pi_{t}E_{t}^{\mathrm{net}}}}+\underset{J_{2}\left(T_{t}^{\mathrm{in}}\right)}{\underset{︸}{\epsilon \sum_{t\in T}\left|T_{t}^{\mathrm{in}}-T^{\mathrm{set}}\right|}}.$$

In Equation (1), $J_{1}\left(E_{t}^{\mathrm{net}}\right)$ is the total electricity cost, calculated under the TOU price $\pi_{t}$ and the net energy consumption $E_{t}^{\mathrm{net}}$ at time *t*. Furthermore, $E_{t}^{\mathrm{net}}$ is written in terms of the energy consumption for the controllable/uncontrollable appliances and the predicted PV generation output. $J_{2}\left(T_{t}^{\mathrm{in}}\right)$ is the total penalty amount involving the consumer discomfort cost. Discomfort implies a deviation of the preferred consumer temperature $T^{\mathrm{set}}$ from the indoor temperature $T_{t}^{\mathrm{in}}$. ϵ is a penalty term for the consumer discomfort cost. A larger ϵ leads to a smaller $J_{2}\left(T_{t}^{\mathrm{in}}\right)$, thereby providing the consumer with decreasing discomfort, while resulting in less energy savings. The value of ϵ can be determined by the HEMS operator to satisfy the consumer preferred comfort level at the expense of the consumer electricity bill. The following subsections demonstrate the equality and inequality constraints for the HEMS optimization problem.

#### 3.2.2. Net Power Consumption

Equation (2) is the constraint on the net energy consumption; that is, the difference between the total consumption of all appliances $\sum_{a\in A}E_{a,t}$ and the predicted PV generation output ${\hat{E}}_{t}^{\mathrm{PV}}$. In Equation (3), the total consumption of all appliances in Equation (2) is decomposed into four different types of reducible appliances ($a\in A_{r}^{c}$), shiftable appliances with a non-interruptible load ($a\in A_{s}^{c,NI}$), shiftable appliances with an interruptible load ($a\in A_{s}^{c,I}$), and uncontrollable appliances ($a\in A^{uc}$):

(2) $$\begin{array}{cc} E_{t}^{\mathrm{net}} & =\sum_{a\in A}E_{a,t}-{\hat{E}}_{t}^{\mathrm{PV}}, \end{array}$$

(3) $$\begin{array}{cc} \sum_{a\in A}E_{a,t} & =\sum_{a\in A_{r}^{c}}E_{a,t}+\sum_{a\in A_{s}^{c,NI}}E_{a,t}+\sum_{a\in A_{s}^{c,I}}\left(E_{a,t}^{\mathrm{ch}}-E_{a,t}^{\mathrm{dch}}\right)+\sum_{a\in A^{uc}}E_{a,t}. \end{array}$$

#### 3.2.3. Operating Characteristics for Controllable Appliances

For a reducible appliance $a\in A_{r}^{c}$, Equation (4) is the constraint for the temperature dynamics of the reducible appliance (for example, air conditioner) at time *t* ($T_{t}^{\mathrm{in}}$), which is expressed in terms of $T_{t-1}^{\mathrm{in}}$ at time t-1, the predicted outdoor temperature at time t-1 (${\hat{T}}_{t-1}^{\mathrm{out}}$), the energy consumption of the reducible appliances ($E_{a,t}$), and the environmental parameters (α,β) specifying the indoor thermal condition. Equation (5) presents the range of consumer preferred indoor temperatures with $T^{\min}$ and $T^{\max}$. The energy consumption capacity for the reducible appliance is limited with $E_{a}^{\min}$ and $E_{a}^{\max}$ in (6):

(4) $$\begin{array}{cc} T_{t}^{\mathrm{in}} & =T_{t-1}^{\mathrm{in}}+\alpha \left({\hat{T}}_{t-1}^{\mathrm{out}}-T_{t-1}^{\mathrm{in}}\right)+\beta E_{a,t}, \end{array}$$

(5) $$\begin{array}{cc} T^{\min} & \leq T_{t}^{\mathrm{in}}\leq T^{\max}, \end{array}$$

(6) $$\begin{array}{cc} E_{a}^{\min} & \leq E_{a,t}\leq E_{a}^{\max}. \end{array}$$

Equations (7)–(9) ensure the desired operation of shiftable appliances with a non-interruptible load $a\in A_{s}^{c,NI}$ (for example, a washing machine) with the binary decision variable $b_{a,t}^{c,NI}$: (i) for the stopping period where $\omega_{s}^{\mathrm{pref}}$ and $\omega_{f}^{\mathrm{pref}}$ are the consumer preferred starting and finishing time in Equation (7), (ii) for the operation period of $L_{a}$ hours during a day in Equation (8), and (iii) for a consecutive operation period of $L_{a}$ hours in Equation (9). The energy consumption capacity for the shiftable appliances with a non-interruptible load is described with $E_{a}^{\max}$ in Equation (10):

(7) $$\begin{array}{cc} b_{a,t}^{c,NI} & =0,\;\;\;\;\;\;\;\;\;\;t\in \left[1,\omega_{s}^{\mathrm{pref}}\right)\cup \left(\omega_{f}^{\mathrm{pref}},T\right], \end{array}$$

(8) $$\begin{array}{cc} \sum_{t=\omega_{s}^{\mathrm{pref}}}^{\omega_{f}^{\mathrm{pref}}}b_{a,t}^{c,NI} & =L_{a}, \end{array}$$

(9) $$\begin{array}{cc} \sum_{t=p}^{p+L_{a}-1}b_{a,t}^{c,NI} & \geq \left(b_{p}^{c,NI}-b_{p-1}^{c,NI}\right)L_{a},\;\;\;\forall p\in \left(\omega_{s}^{\mathrm{pref}},\omega_{f}^{\mathrm{pref}}-L_{a}+1\right) \end{array}$$

(10) $$\begin{array}{cc} E_{a,t} & =b_{a,t}^{c,NI}E_{a}^{\max}. \end{array}$$

Equation (11) illustrates the operational dynamics of the state of energy (SOE) for the ESS ($a\in A_{s}^{c,I}$) at the current time *t* in terms of the SOE at the previous time t-1, the charging and discharging efficiency, $\eta_{a}^{\mathrm{ch}}$ and $\eta_{a}^{\mathrm{dch}}$, and the charging and discharging energy, $E_{a,t}^{\mathrm{ch}}$ and $E_{a,t}^{\mathrm{dch}}$, respectively. Equation (12) provides the SOE capacity constraint with $SOE_{a}^{\min}$ and $SOE_{a}^{\max}$ for the ESS. Equations (13) and (14) present the constraints on the charging ($E_{a,t}^{\mathrm{ch}}$) and discharging ($E_{a,t}^{\mathrm{dch}}$) energies of the ESS, respectively, where $b_{a,t}^{c,I}$ represents the binary decision variable that determines the ESS on/off status:

(11) $$\begin{array}{cc} SOE_{a,t}=SOE_{a,t-1} & +\eta_{a}^{\mathrm{ch}}E_{a,t}^{\mathrm{ch}}-\frac{E_{a,t}^{\mathrm{dch}}}{\eta_{a}^{\mathrm{dch}}}, \end{array}$$

(12) $$\begin{array}{cc} SOE_{a}^{\min}\leq & SOE_{a,t}\leq SOE_{a}^{\max}, \end{array}$$

(13) $$\begin{array}{cc} E_{a}^{\mathrm{ch},\min}b_{a,t}^{c,I}\leq & E_{a,t}^{\mathrm{ch}}\leq E_{a}^{\mathrm{ch},\max}b_{a,t}^{c,I}, \end{array}$$

(14) $$\begin{array}{cc} E_{a}^{\mathrm{dch},\min}\left(1-b_{a,t}^{c,I}\right)\leq & E_{a,t}^{\mathrm{dch}}\leq E_{a}^{\mathrm{dch},\max}\left(1-b_{a,t}^{c,I}\right). \end{array}$$

Finally, the MINLP-based HEMS optimization problem above can be converted into an MILP optimization problem by means of the linearization of the nonlinear objective function $J_{2}\left(T_{t}^{\mathrm{in}}\right)$ as follows:

(15) $$\begin{array}{cc} \Delta T_{t} & =|T_{t}^{\mathrm{in}}-T^{\mathrm{set}}|, \end{array}$$

(16) $$\begin{array}{cc} \Delta T_{t} & \geq T_{t}^{\mathrm{in}}-T^{\mathrm{set}}, \end{array}$$

(17) $$\begin{array}{cc} \Delta T_{t} & \geq T^{\mathrm{set}}-T_{t}^{\mathrm{in}}. \end{array}$$

## 4. Formulation of RL- and ANN-Based Home Energy Management

### 4.1. Home Energy Management via Q-Learning

**Figure 2:**
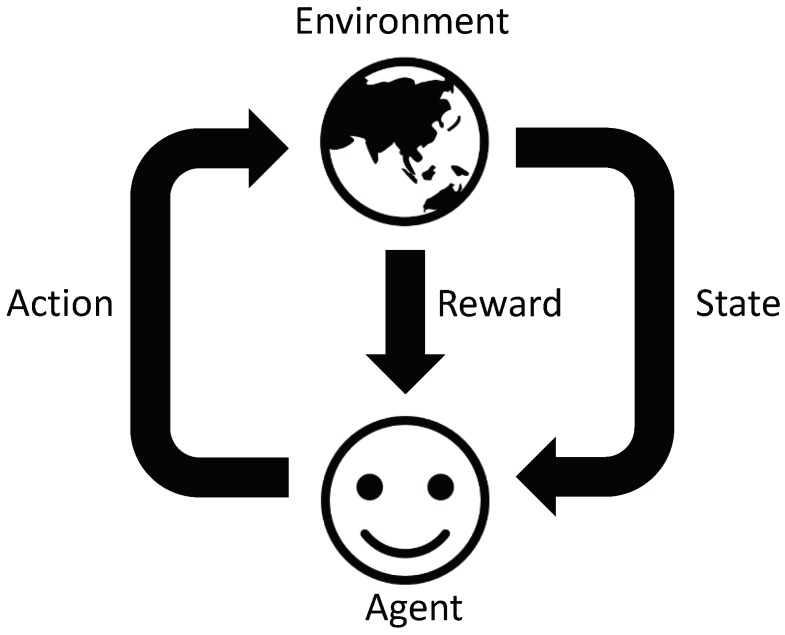
Conceptual architecture of reinforcement learning (RL).

RL is one of the main ML techniques for optimal decision-making in a non-deterministic environment. As illustrated in Figure 2, while an agent interacts with an environment, the agent learns the type of action depending on the state of the environment, and sends the learned action to the environment. The environment then returns a reward along with the new state of the environment to the agent. This learning process continues until the agent maximizes the total cumulative rewards received from the environment. A policy is defined as the manner in which the agent acts from a specific state, and the primary goal of the agent is to determine the optimal policy that maximizes the reward. In this study, we assume that the environment is described by a Markov decision process, in which the agent state transition relies only on the present state, along with the action selected in the present state, without considering all past states and actions.

Q-learning is one of the representative RL techniques for determining the optimal policy $\nu^{*}$ of a decision-making problem. The general process of Q-learning calculates a Q-value $Q(s_{t},a_{t})$ of a pair of state $s_{t}$ and action $a_{t}$ at a discrete time *t* and updates the Q-value towards the maximum total rewards using the following Bellman equation:

(18) $$Q_{\nu^{*}}^{*}\left(s_{t},a_{t}\right)=r\left(s_{t},a_{t}\right)+\gamma \max Q\left(s_{t+1},a_{t+1}\right).$$

In Equation (18), based on the optimal policy $\nu^{*}$, the optimal Q-value $Q_{\nu^{*}}^{*}\left(s_{t},a_{t}\right)$ is obtained by the summation of the present reward $r(s_{t},a_{t})$ and maximum discounted future reward $\gamma \max Q(s_{t+1},a_{t+1})$ where γ∈[0,1] represents a discounting factor that explains the relative importance of the present and future rewards. As the discounting factor γ decreases, the agent becomes short-sighted because it focuses increasingly on the present reward. However, a larger γ enables the agent to focus increasingly on the future reward and thus become far-sighted. The value of γ can be tuned by the system operator using Q-learning to balance the present and future rewards.

Whenever the Q-value $Q(s_{t},a_{t})$ is updated with a specific pair of state and action at time *t*, $Q(s_{t},a_{t})$ is saved in the state-action table, namely the Q-value table. The agent selects its action using the Q-value table at every time *t*, and the element (Q-value) in the Q-value table associated with the selected pair of state and action is updated using the following Bellman equation:

(19) $$Q\left(s_{t},a_{t}\right)\leftarrow \left(1-\theta \right)Q\left(s_{t},a_{t}\right)+\theta \left[r\left(s_{t},a_{t}\right)+\gamma \max Q\left(s_{t+1},a_{t+1}\right)\right].$$

In Equation (19), θ∈[0,1] represents the learning rate that determines the extent to which the new Q-value overrides the old one. With θ=0, the agent learns nothing and uses only the past Q-value without exploration in the Q-learning process. However, with θ=1, the agent updates its Q-value using only the present reward and maximum discounted future reward without exploitation. Similar to the selection of γ, a trade-off between exploration and exploitation can be determined by the system operator through setting the value of θ in [0,1]. Finally, by updating $Q(s_{t},a_{t})$ in an iterative manner using Equation (19), the Q-value will become increasingly larger, and the agent will obtain the optimal policy $\nu^{*}$ with the largest Q-value, as follows:

(20) $$\nu^{*}=\arg\max Q\left(s_{t},a_{t}\right).$$

In this study, the aforementioned Q-learning method is applied to an individual appliance (for example, air conditioner, washing machine, or ESS) to calculate the optimal operation schedule of appliances in a smart home with a PV system and an ESS, which consequently results in the reduction of the consumer electricity bill within the consumer preferred appliance scheduling and comfort level. A detailed illustration of the state, action, and reward for the proposed Q-learning approach is provided in the following three subsections.

#### 4.1.1. State Space

We consider the situation in which the proposed Q-learning algorithm is executed for 24 h with a 1 h scheduling resolution. For ∀t=1,…,24, the state spaces of the washing machine (WM), air conditioner (AC), and ESS are expressed as follows, respectively:

(21) $$S^{\mathrm{WM}}=\left\{E_{t}^{\mathrm{WM}}\right\},\;\;S^{\mathrm{AC}}=\left\{E_{t}^{\mathrm{AC}}\right\},\;\;S^{\mathrm{ESS}}=\left\{SOE_{t}^{\mathrm{ESS}}\right\},$$

where the states $E_{t}^{\mathrm{WM}}$, $E_{t}^{\mathrm{AC}}$, and $SOE_{t}^{\mathrm{ESS}}$ are the energy consumption of the WM and AC, and SOE of the ESS, respectively, at time *t*.

#### 4.1.2. Action Space

The optimal action for each appliance depends on the environment of the agent, including the present state, as defined in Section 4.1.1. The action spaces of the WM, AC, and ESS are illustrated as follows:

(22) $$\begin{array}{cc} A^{\mathrm{WM}} & =\{\mathrm{On},\mathrm{Off}\}, \end{array}$$

(23) $$\begin{array}{cc} A^{\mathrm{AC}} & =\{0,\Delta E^{\mathrm{AC}},2\Delta E^{\mathrm{AC}},\ldots ,8\Delta E^{\mathrm{AC}},9\Delta E^{\mathrm{AC}}\}, \end{array}$$

(24) $$\begin{array}{cc} A^{\mathrm{ESS}} & =\{-4\Delta E^{\mathrm{ESS}},-3\Delta E^{\mathrm{ESS}},-2\Delta E^{\mathrm{ESS}},-1\Delta E^{\mathrm{ESS}},0,1\Delta E^{\mathrm{ESS}},2\Delta E^{\mathrm{ESS}},3\Delta E^{\mathrm{ESS}},4\Delta E^{\mathrm{ESS}}\}. \end{array}$$

In Equation (22), the WM agent performs the binary action {On, Off}. With the ‘On’ action, the WM agent turns on the WM, which consumes a constant energy ($E^{\mathrm{WM},\max}$), whereas the WM agent turns off the WM with the ‘Off’ action. The action for the AC agent is discretized into 10 levels of AC energy consumption in Equation (23) where $\Delta E^{\mathrm{AC}}$ represents an energy consumption unit of the AC. Similar to the action for the AC agent, the discrete set of actions for the ESS agent is defined with an energy unit of ESS $\Delta E^{\mathrm{ESS}}$ in Equation (24). These discretized actions are categorized into discharging and charging actions, corresponding to $\left\{-4\Delta E^{\mathrm{ESS}},-3\Delta E^{\mathrm{ESS}},-2\Delta E^{\mathrm{ESS}},-1\Delta E^{\mathrm{ESS}}\right\}$ and $\left\{1\Delta E^{\mathrm{ESS}},2\Delta E^{\mathrm{ESS}},3\Delta E^{\mathrm{ESS}},4\Delta E^{\mathrm{ESS}}\right\}$, respectively. The proposed algorithm calculates an hourly energy consumption schedule for the appliances for the next 24 h. Given the state and action sets above, the Q-value tables for the WM, AC, and ESS agents are illustrated using the $\left|T|\;\times \;\right|A^{\mathrm{WM}}|$, $\left|T|\;\times \;\right|A^{\mathrm{AC}}|$, and $\left|T|\;\times \;\right|A^{\mathrm{ESS}}|$ matrices, with |T| = 24, $|A^{\mathrm{WM}}|\;=\;2$, $|A^{\mathrm{AC}}|\;=\;10$, and $|A^{\mathrm{ESS}}|\;=\;9$, respectively. In this case, |A| is the cardinality of the set A (that is, the number of elements in A).

#### 4.1.3. Reward

The reward function for each appliance agent is formulated as the sum of the negative electric cost and negative dissatisfaction cost associated with the consumer preferred comfort and appliance operation characteristics. The comprehensive reward $r^{\mathrm{Total}}$ for the HEMS is defined as

(25) $$r^{\mathrm{Total}}=r_{t}^{\mathrm{WM}}+r_{t}^{\mathrm{AC}}+r_{t}^{\mathrm{ESS}}.$$

In Equation (25), the three reward functions $r_{t}^{\mathrm{WM}}$, $r_{t}^{\mathrm{AC}}$, and $r_{t}^{\mathrm{ESS}}$ aim to evaluate the HEMS performance in terms of: (i) the electric cost and consumer undesired operation of the WM, (ii) the electric cost and consumer thermal discomfort of the AC, and (iii) the electric cost and energy underutilization owing to overcharging and undercharging of the ESS.

Firstly, the reward function for the WM agent is expressed as

(26) $$\begin{array}{c} r_{t}^{\mathrm{WM}}=\left\{\begin{array}{cc} -\left[\pi_{t}E_{t}^{\mathrm{WM}}+\bar{\delta }\left(\omega_{s}^{\mathrm{pref}}-t\right)\right],\;\; & \mathrm{if}\;t<\omega_{s}^{\mathrm{pref}},\\ -\left[\pi_{t}E_{t}^{\mathrm{WM}}+\underline{\delta }\left(t-\omega_{f}^{\mathrm{pref}}\right)\right], & \mathrm{if}\;t>\omega_{f}^{\mathrm{pref}},\\ -\pi_{t}E_{t}^{\mathrm{WM}}, & \mathrm{otherwise}, \end{array}\right. \end{array}$$

where $\omega_{s}^{\mathrm{pref}}$ and $\omega_{f}^{\mathrm{pref}}$ are the consumer preferred starting and finishing times of the WM, respectively, while $\bar{\delta }$ and $\underline{\delta }$ are the penalties for early and late operation, respectively, compared to the consumer preferred operation interval. A dissatisfaction cost is added to the reward function with a negative value if the WM agent schedules the WM energy consumption before $\omega_{s}^{\mathrm{pref}}$ or after $\omega_{f}^{\mathrm{pref}}$; otherwise, the reward function includes only a negative electric cost.

The reward function for the AC agent is defined as

(27) $$\begin{array}{c} r_{t}^{\mathrm{AC}}=\left\{\begin{array}{cc} -\left[\pi_{t}E_{t}^{\mathrm{AC}}+\kappa \left(T^{\min}-T_{t}^{\mathrm{in}}\right)\right],\;\; & \mathrm{if}\;T_{t}^{\mathrm{in}}<T^{\min},\\ -\left[\pi_{t}E_{t}^{\mathrm{AC}}+\kappa \left(T_{t}^{\mathrm{in}}-T^{\max}\right)\right], & \mathrm{if}\;T_{t}^{\mathrm{in}}>T^{\max},\\ -\pi_{t}E_{t}^{\mathrm{AC}}, & \mathrm{otherwise}, \end{array}\right. \end{array}$$

where κ is the penalty for the consumer thermal discomfort. The dissatisfaction cost is defined as the deviation of the consumer preferred temperature $T_{t}^{\mathrm{in}}$ from $T^{\min}$ and $T^{\max}$, and it is considered as the reward with a negative sign only if $T_{t}^{\mathrm{in}}$ deviates from the range of [$T^{\min}$, $T^{\max}$].

Finally, the reward function for the ESS agent consists of a negative electric cost and negative energy underutilization cost, as follows:

(28) $$\begin{array}{c} r_{t}^{\mathrm{ESS}}=\left\{\begin{array}{cc} -\left[\pi_{t}E_{t}^{\mathrm{ESS}}+\bar{\tau }\left(SOE_{t}-SOE^{\max}\right)\right],\;\; & \mathrm{if}\;SOE_{t}>SOE^{\max},\\ -\left[\pi_{t}E_{t}^{\mathrm{ESS}}+\underline{\tau }\left(SOE^{\min}-SOE_{t}\right)\right], & \mathrm{if}\;SOE_{t}<SOE^{\min},\\ -\pi_{t}E_{t}^{\mathrm{ESS}}, & \mathrm{otherwise}, \end{array}\right. \end{array}$$

where $\bar{\tau }$ and $\underline{\tau }$ are the penalties for the ESS overcharging and undercharging, respectively. In this case, energy underutilization of the ESS occurs if the SOE becomes lower than $SOE^{\min}$ (undercharging) or greater than $SOE^{\max}$ (overcharging), and it is considered as a reward term, along with the electric cost during the ESS underutilization stage.

### 4.2. Prediction of Indoor Temperature via ANN

In this study, we consider the situation in which the HEMS schedules the AC energy consumption based on the indoor and outdoor temperature with the consumer preferred thermal conditions. Traditionally, the HEMS calculates the current indoor temperature using an approximated equation (that is, the equivalent thermal parameters (ETP) model Equation (4) in Section 3.2.3 in terms of the previous indoor and current outdoor temperature, AC energy consumption, and indoor thermal characteristics). In this subsection, in contrast to the aforementioned model-based approach for the indoor temperature prediction, we propose an ANN-based method for predicting the indoor temperature associated with the AC energy consumption.

In the proposed ANN model, the AC agent learns the extent to which the AC energy consumption affects the current indoor temperature, which implies the estimation of the function *f* that illustrates the relationship between the indoor temperature and AC energy consumption, as follows:

(29) $$\hat{f}\left(T_{t-1}^{\mathrm{in}},T^{\min},T^{\max},{\hat{T}}_{t}^{\mathrm{out}},E_{t}^{\mathrm{AC}}\right)=T_{t}^{\mathrm{in}},$$

where $\hat{f}$ is the approximated function that explains the relationship between the input data from the ETP model in Section 3.2.3), such as the previous indoor temperature ($T_{t-1}^{\mathrm{in}}$), consumer’s preferred indoor thermal conditions ($T^{\min}$, $T^{\max}$), weather forecasting (${\hat{T}}_{t}^{\mathrm{out}}$), and AC energy consumption ($E_{t}^{\mathrm{AC}}$) and the output for the predicted current indoor temperature.

**Figure 3:**
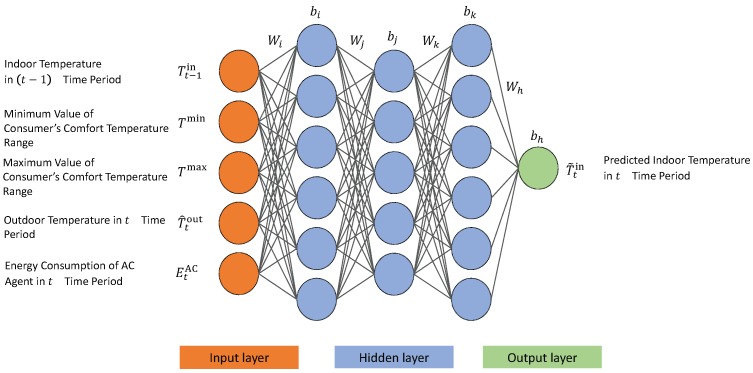
Architecture of the proposed artificial neural network (ANN) model.

As illustrated in Figure 3, the proposed ANN model consists of one input data layer with five neurons, three hidden layers with seventeen neurons, and one output layer with one neuron. Each layer calculates the weighted sum of the input vector and a constant bias $b_{i}$ with a weight $W_{i}$, and the weighted sum is transferred to the following layer by means of the transfer function. In this study, Rectified Linear Unit (ReLu) function is used as a transfer function [34]. Moreover, the Adam optimization algorithm [35] is employed to train the proposed ANN model, and the learning rate of the optimization algorithm is set to 0.005.

The temperature prediction function approximated by the proposed ANN is fed into the Q-learning module for the AC agent, as illustrated in Section 4.1. This approximated model enables the AC agent to calculate the dissatisfaction cost more precisely and determine the optimal energy consumption schedule more efficiently during the Q-learning process.

**Figure 4:**
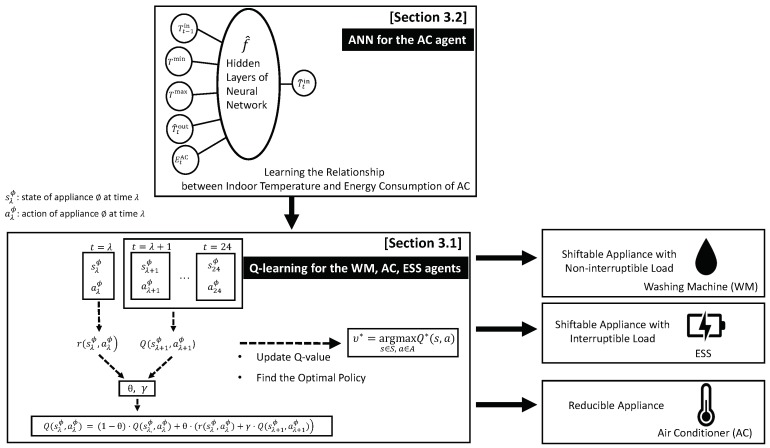
Overall architecture of the proposed HEMS algorithm using Q-learning and ANN.

Finally, the HEMS with the PV system, ESS, and home appliances learn the energy management policies that optimize the electricity bill and consumer comfort level using Algorithm 1. The HEMS receives the hour-ahead indoor temperature, consumer preferred indoor temperature range, predicted outdoor temperature, and AC energy consumption ($E_{t}^{\mathrm{AC}}$), and uses the ANN to predict the current indoor temperature. Afterwards, the proposed Q-learning is initiated to schedule the optimal energy consumption of the appliances and ESS charging/discharging. Figure 4 illustrates the proposed Q-learning- and ANN-based framework for optimal control of the home appliances and ESS.

**Algorithm 1:** Q-learning-based energy management of smart home with PV system, ESS, and home appliances.   **_1_**  Initialize each appliance’s energy demand, dissatisfaction parameters, and Q-learning parameters    **_2_**  %%Learning with ANN for temperature prediction of AC agent    **_3_**  Indoor temperature at time period t-1 → $T_{t-1}^{\mathrm{in}}$
   **_4_**  Minimum and maximum value of consumer’s comfort temperature range → $T^{\min}$, $T^{\max}$
   **_5_**  Predicted outdoor temperature at time *t* → ${\hat{T}}_{t}^{\mathrm{out}}$
   **_6_**  Energy consumption of AC agent at time *t* → $E_{t}^{\mathrm{AC}}$
   **_7_**  Predicted indoor temperature at time *t* → $T_{t}^{\mathrm{in}}$
   **_8_**  Learning process with ANN and approximate the temperature prediction model $\hat{f}$
   **_9_**  $T_{t}^{\mathrm{in}}=\hat{f}\left(T_{t-1}^{\mathrm{in}},T^{\max},T^{\min},{\hat{T}}_{t}^{\mathrm{out}},E_{t}^{\mathrm{AC}}\right)$
  **_10_**  Initialize Q-value of each agent  
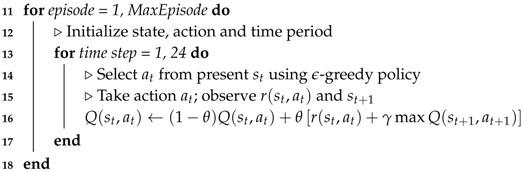

  **_19_**  Find optimal policy with largest Q-value

## 5. Numerical Examples

### 5.1. Simulation Setup

**Figure 5:**
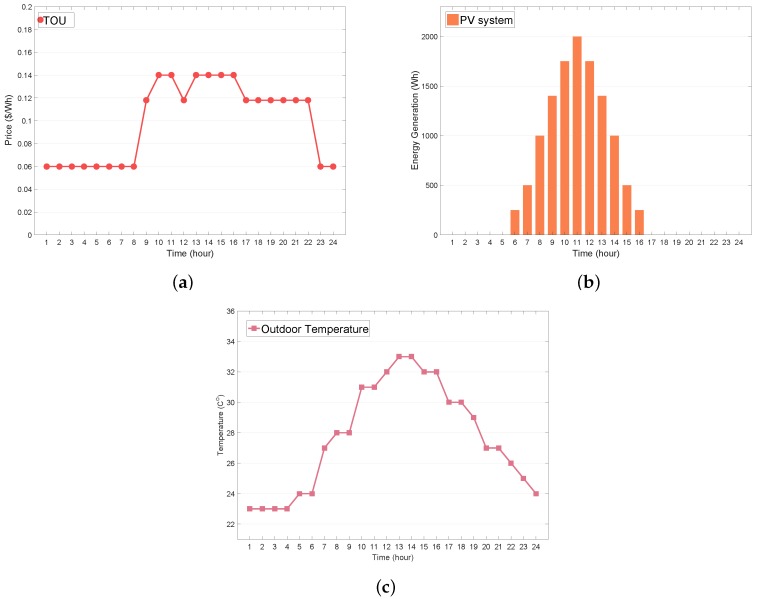
Profiles of electricity price and weather. (**a**) time-of-use (TOU) price; (**b**) solar photovoltaic (PV) generation; (**c**) outdoor temperature.

We considered the situation of a household with two major home appliances (AC and WM), and an ESS that can be controlled by the HEMS under the TOU tariff, as illustrated in Figure 5a. The simulations were carried out for 24 h with a 1 h scheduling resolution. It was assumed that the predicted PV generation energy ${\hat{E}}_{t}^{\mathrm{PV}}$ in Figure 5b and outdoor temperature ${\hat{T}}_{t-1}^{\mathrm{out}}$ in Figure 5c could be obtained accurately. The maximum energy consumptions of the AC, WM, and aggregated uncontrollable appliances were 3000, 500, and 1700 Wh, respectively. The consumer comfortable temperature range was assumed to be (23 °C, 25 °C). The consumer preferred temperature $T^{\mathrm{set}}$ is 24 °C, and the penalties ϵ (MILP-based HEMS) and κ (RL-based HEMS) for the consumer thermal discomfort were both 100. The parameters α and β, which represent the AC thermal characteristics, were set to 0.8 and −0.02, respectively. The allowable operating period for the WM was (6:00 a.m., 10:00 p.m.), and the consecutive operation time was 2 h. The maximum charging and discharging capacities, $E^{\mathrm{ch},\max}$ and $E^{\mathrm{dch},\max}$, for the ESS were both 4000 Wh, while the initial, minimum, and maximum SOE values were 2400, 800, and 4000 Wh, respectively. In the action space, the energy consumption units, $\Delta E^{\mathrm{AC}}$ and $\Delta E^{\mathrm{ESS}}$, for the air conditioner and ESS are 40 Wh and 150 Wh, respectively. For the reward function, the penalties for the dissatisfaction cost of the WM and ESS were $\bar{\delta }=50$, $\underline{\delta }=50$, $\bar{\tau }=50$, and $\underline{\tau }=50$, respectively. The parameter ϵ of the ϵ-greedy policy for exploration and exploitation was set to 0.1. The learning rate θ and discounting factor γ in the Bellman equation were set to 0.1 and 0.9, respectively. The proposed algorithm was implemented on a computer (AMD Ryzen 7 2700X 8-core CPU (China) clocking at 3.70 Hz and 32 GB of RAM), using the optimization toolbox in MATLAB R2018a (MILP optimization) (MathWorks, Natick, MA, USA) and Python (Q-learning and ANN).

### 5.2. Performance of the Proposed RL-Based HEMS Algorithm

**Figure 6:**
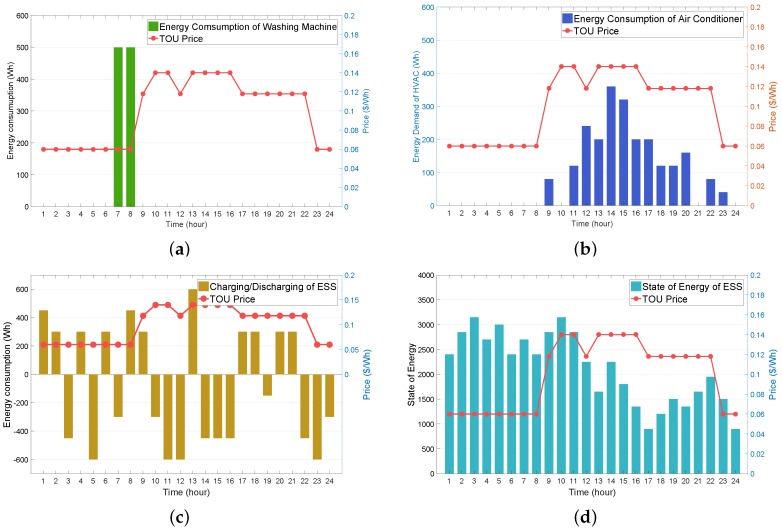
RL-based day-ahead operating schedule of appliance under TOU pricing tariff. (**a**) energy consumption of washing machine (WM); (**b**) energy consumption of air conditioner (AC); (**c**) charging and discharging of energy storage system (ESS); (**d**) state of energy (SOE) of ESS.

In this subsection, we present the simulation of the algorithm for the proposed RL-based HEMS, and verify the energy consumption schedule of the controllable appliances and ESS charging/discharging schedule. Figure 6a illustrates the energy consumption schedule calculated by the WM agent. It can be observed from Figure 6a that, given the consumer preferred operation period (6:00 a.m., 10:00 p.m.) with two consecutive operation hours (L=2), the optimal operation schedule for the washing machine was selected as (7:00 a.m., 8:00 a.m.). This scheduling policy is considered as optimal because a washing machine operates at the lowest TOU price, which in turn reduces the electricity bill, while satisfying the consumer preference. Figure 6c,d illustrate the charging/discharging and SOE schedules for the ESS, respectively. Similarly to the result in Figure 6a, it can be observed from Figure 6c that, in general, the charging (positive energy consumption) of the ESS occurred at low TOU prices, whereas the discharging (negative energy consumption) of the ESS occurred at high TOU prices, thereby leading to consumer energy savings. Furthermore, it can be observed from Figure 6d that, as the price increased, the SOE decreased, and vice versa. This is because a higher price results in the ESS discharging and hence the SOE decreases. For example, the ESS discharged at 4:00 p.m. with the highest price in Figure 6c, which led to a decreasing SOE from 4 to 5:00 p.m. in Figure 6d. However, the ESS charged at 5:00 p.m. owing to the decreasing price, and, consequently, the SOE increased from 5 to 6:00 p.m. Figure 6b illustrates the AC energy consumption schedule. Compared to the results of the WM and ESS agents, we observe from this figure that a high (or low) price did not always enable the AC agent to decrease (or increase) the AC energy consumption. This is owing to the fact that the AC agent considers the consumer thermal comfort as well as the electricity bill saving in the reward function.

In the Q-learning process for the AC agent, a higher penalty κ for the consumer thermal discomfort led to a lower energy saving and vice versa. Regarding the trade-off between the energy saving and consumer comfort in terms of κ, HEMS operators may adaptively adjust the penalty κ to situations in which the consumer aims to save more on the electricity costs or maintain a more comfortable environment. A detailed assessment of the impact of the penalty for the AC agent on the proposed algorithm is presented in the following subsection.

### 5.3. Impact of Different Parameters in Reward Function on the Proposed Algorithm

**Figure 7:**
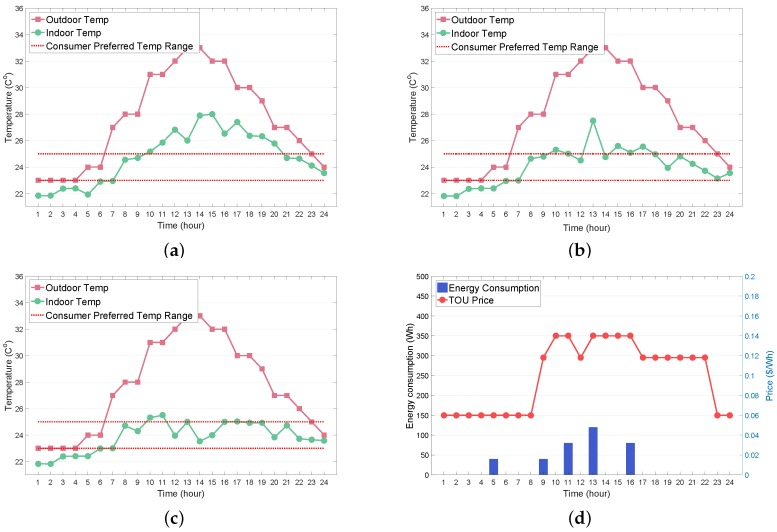

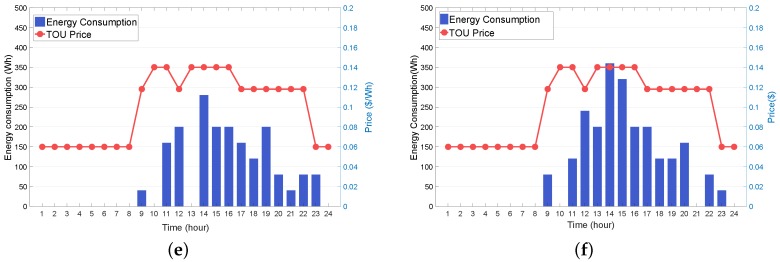
Impact of different penalties (κ) for AC scheduling on indoor temperature ($T_{t}^{\mathrm{in}}$) and energy consumption ($E_{a,t}$). (**a**) $T_{t}^{\mathrm{in}}$ with κ=10; (**b**) $T_{t}^{\mathrm{in}}$ with κ=50; (**c**) $T_{t}^{\mathrm{in}}$ with κ=100; (**d**) $E_{a,t}$ with κ=10; (**e**) $E_{a,t}$ with κ=50; (**f**) $E_{a,t}$ with κ=100.

In this subsection, we investigate the effects of the different penalties κ and preferred operating time intervals $\left[\omega_{s}^{\mathrm{pref}},\;\omega_{f}^{\mathrm{pref}}\right]$ in the reward function for the AC and WM on the performance of the AC and WM agents. Figure 7a–c illustrate the impact of varying κ values (κ=10,50,100) on the indoor temperature $T_{t}^{\mathrm{in}}$ at any time period *t* given an outdoor temperature $T_{t}^{\mathrm{out}}$. As indicated in Figure 7a, when κ was set to 10, the indoor temperature exhibited a significant deviation from the consumer preferred temperature range (23 °C, 25 °C) in most time periods. It can be observed from Figure 7b,c that an increasing κ caused the indoor temperature to deviate less from the consumer preferred temperature range. This observation derives from the fact that the AC agent with an increasing κ aims to update the Q-value toward maximizing the satisfaction cost of the consumer indoor thermal condition at the expense of the consumer electricity bill. The trade-off between the energy saving and consumer comfort in terms of κ is verified by the comparison of Figure 7a–c and Figure 7d–f. As expected, it is observed from Figure 7d–f that, as the value of κ increased, the AC energy consumption also increased to maintain the consumer comfort.

**Figure 8:**
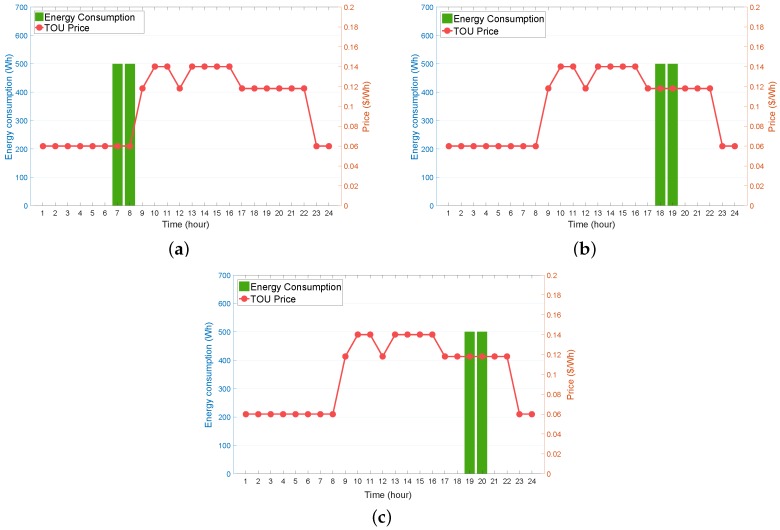
Impact of different preferred operating time interval $\left[\omega_{s}^{\mathrm{pref}},\omega_{f}^{\mathrm{pref}}\right]$ for WM scheduling on energy consumption ($E_{a,t}$). (**a**) (6:00 a.m., 10:00 p.m.); (**b**) (12:00 p.m., 10:00 p.m.); (**c**) (5:00 p.m., 10:00 p.m.).

Figure 8a–c illustrate the effects of varying preferred operating time intervals of the WM on the WM energy consumption. The results from these figures correspond to three different operating time intervals, namely (6:00 a.m., 10:00 p.m.) (Figure 8a), (12:00 p.m., 10:00 p.m.) (Figure 8b), and (5:00 p.m., 10:00 p.m.) (Figure 8c), where the finishing time $\omega_{f}^{\mathrm{pref}}$ in each time interval was identically set to 10 p.m. with varying starting time $\omega_{s}^{\mathrm{pref}}$ ($\omega_{s}^{\mathrm{pref}}$ = 6:00 a.m., 12:00 p.m., and 5:00 p.m.). It can be observed from Figure 8a–c that the optimal operating schedule of the WM was selected at the time periods with the lowest TOU price within the preferred operating time interval. This observation confirms that the WM agent could always determine the optimal policy to minimize the electricity bill successfully, provided that the consumer preferred operating time interval changed.

### 5.4. Impact of ANN on AC Agent Performance

**Figure 9:**
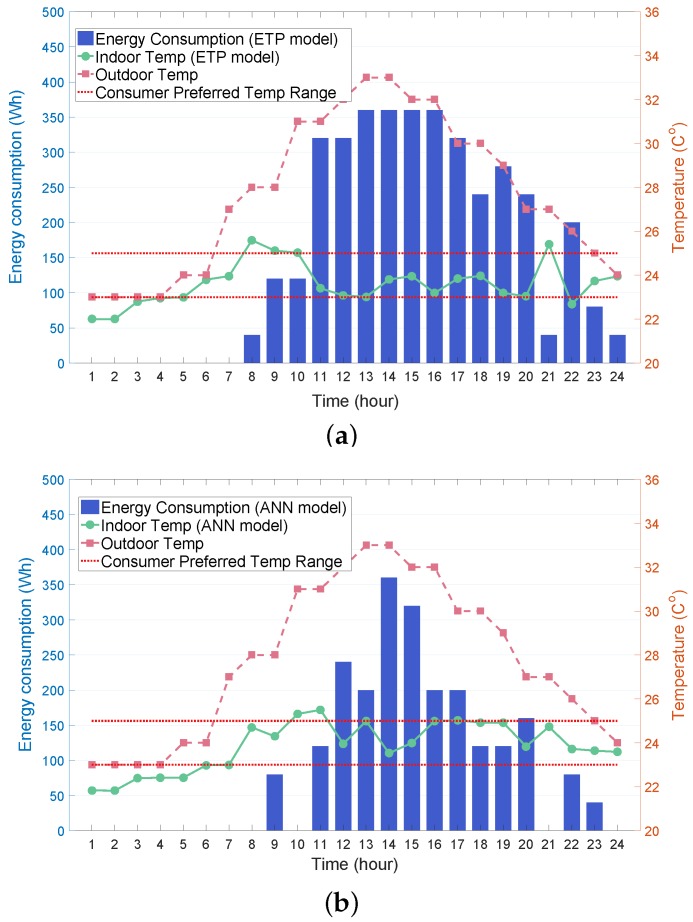
RL-based day-ahead energy consumption schedule of air conditioner through indoor temperature prediction using: (**a**) equivalent thermal parameters (ETP) model; (**b**) ANN model.

In this subsection, we study the effect of the indoor temperature prediction using the ANN model proposed in Section 4.2 on the performance of the proposed RL-based algorithm. Figure 9a,b compare the AC energy consumption and indoor temperature obtained by the Q-learning process between the ETP model and ANN models. This comparison verifies that the ANN model (Figure 9b) required less energy consumption than the ETP model (Figure 9a). This is because the ANN assisted the AC agent in learning the relationship between the indoor temperature and AC energy consumption more accurately, and, consequently, the AC agent determined the optimal policy to achieve greater energy savings. Furthermore, the proposed ANN approach is beneficial for HEMS operators in the following manner. During the RL-based HEMS execution, the HEMS operators use only various data types for the AC energy consumption, consumer preference, and weather forecasting without explicitly relying on the model-based ETP equation with fixed environmental parameters (α and β in Equation (4)) for the indoor thermal conditions. Therefore, the HEMS operators do not need to tune these parameters, even though the household environment varies.

### 5.5. Performance Comparison between MILP- and RL-Based HEMS

**Figure 10:**
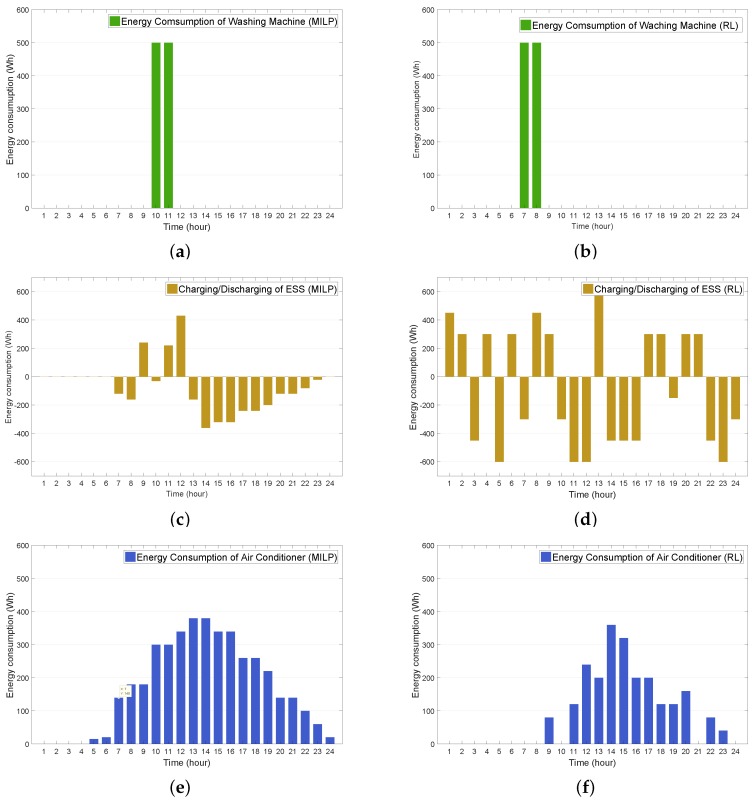
Comparison of energy consumption and charing/discharging energy between mixed-integer linear programming (MILP) and RL methods. (**a**) WM with MILP; (**b**) WM with RL; (**c**) ESS with MILP; (**d**) ESS with RL; (**e**) air conditioner (AC) with MILP; (**f**) AC with RL.

In this subsection, we compare the performance of the proposed RL-based HEMS algorithm with that of the MILP-based HEMS algorithm. Figure 10a,b illustrate the AC energy consumption using the MILP model and RL model, respectively. It can be observed from these figures that the operation periods for the WM were scheduled for (10 A.M., 11 A.M.) in the MILP model and (7 A.M., 8 A.M.) in the RL model. Considering the TOU price, this shift in the WM operation periods provide the consumer with an energy saving of $80 with the RL approach, where the electric costs for the MILP and RL models were $140 and $60, respectively. The comparison results of the ESS charging and discharging schedule between between the MILP and RL models are presented in Figure 10c,d. Compared to the charing and discharging schedule for the MILP model illustrated in Figure 10c, it is observed from Figure 10d that the ESS charged and discharged a significant amount of energy, consequently achieving an energy saving of $94.09 where the electric costs for the MILP model and RL models were −$193.91 and −$288, respectively. Moreover, it can be observed from Figure 10e,f that the RL approach reduced the AC energy consumption more than the MILP approach, consequently leading to a reduction in the electric cost of $228.28, where the electric costs for the MILP and RL models were $462.62 and $234.34, respectively.

**Figure 11:**
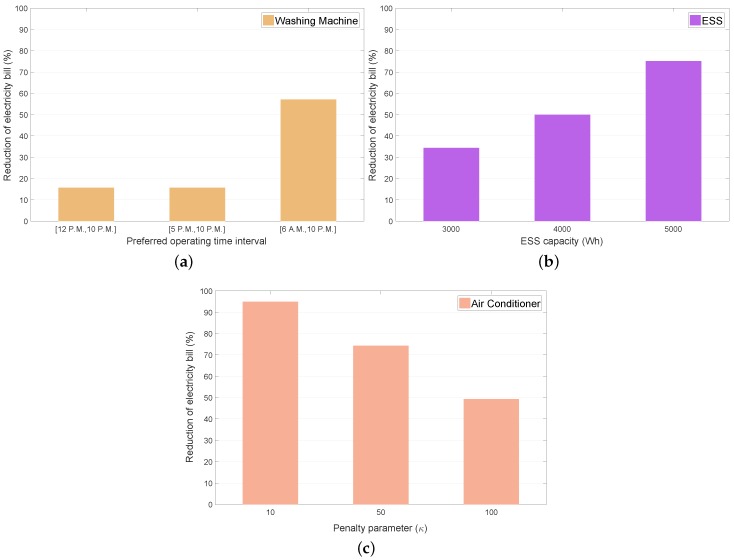
Reduction in electricity bill with different operating conditions of appliances. (**a**) WM under varying preferred operating time intervals; (**b**) ESS under varying capacity; (**c**) AC under varying penalty parameters.

Figure 11a–c indicate a relative reduction in the total electricity bill in the RL model with varying parameters according to the following index:

(30) $$\frac{X^{\mathrm{bill},\mathrm{MILP}}-X_{p}^{\mathrm{bill},\mathrm{RL}}}{X^{\mathrm{bill},\mathrm{MILP}}}\times 100\left(\%\right),$$

where $X^{\mathrm{bill},\mathrm{MILP}}$ is the total electricity bill in the RL model using the MILP and $X_{p}^{\mathrm{bill},\mathrm{RL}}$ is the total electricity bill using the RL where *p* represents a parameter including the preferred operating time interval of the WM, ESS capacity, and penalty for the consumers preferred indoor thermal conditions associated with the AC operation. It can be observed from these figures that the RL method could achieve greater energy savings than the MILP model under the situation with varying parameters.

The results from Figure 11a indicate that the longer preferred operating time interval of the WM enabled the WM agent to select the operating intervals efficiently, and the consumer to obtain greater energy savings. As expected, as the ESS capacity increased, the ESS agent conducted additional energy charging and discharging to reduce the electricity bill, which is verified by Figure 11b. It is also observed from Figure 11c that the smaller penalty for the consumer preferred indoor thermal condition led to a greater energy savings, where the AC agent minimized the WM electricity cost at the expense of the consumer indoor thermal comfort.

**Figure 12:**
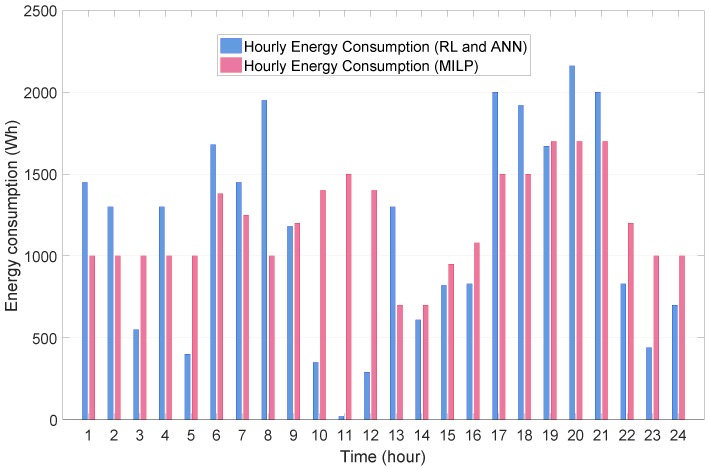
Comparison of hourly energy consumption schedule between MILP method and RL methods.

Finally, Figure 12 compares the total energy consumption every hour between the MILP method and the proposed RL method integrated with the ANN. It can be verified from this figure that the energy consumption using the proposed approach was significantly reduced in the following three time periods: (3:00 a.m., 5:00 a.m.), (10:00 a.m., 12:00 p.m.), and (10:00 p.m., 12:00 a.m.). In this simulation study, the relative electricity bill reduction of the proposed RL method compared to the MILP method using Equation (30) was calculated as 14%.

## 6. Discussion

### 6.1. Wholesale and Retail Electricity Markets under Real-Time Pricing (RTP)

In this study, the proposed HEMS algorithm is executed under the TOU pricing tariff. However, in electric power system operations, there is another pricing tariff such as real-time pricing (RTP). RTP, namely locational marginal pricing, is the core variable to conduct the congestion management in the wholesale and retail electricity markets [36,37]. Recently, a two-stage home energy management algorithm has been developed under distribution locational marginal pricing [38]. In real-time electricity markets, the results in [31] show that the value of RTP can be accurately forecasted by ANN with various types of input data. Therefore, the ANN-based RTP forecasting module can be integrated into the proposed Q-learning framework illustrated in Section 4 to manage the optimal energy consumption of a smart home under the real-time electricity market environment.

### 6.2. Electric Vehicle (EV) Integration

Recent studies have investigated the joint optimization of electric vehicle (EV) and home energy consumption scheduling [4]. However, these studies are limited to model-based SOE constraints without conducting the travel pattern analysis of EV. To resolve this limitation, a key part in our proposed approach would be to analyze the travel pattern of EV using ANN with historical travel data such as arrival and departure times, the number of travels per day, and the travel distance. Then, the modeling of the SOE dynamics of EV could be performed by the EV agent, which learns the charging or discharging action depending on the SOE state of EV similar to the ESS agent process illustrated in Section 4.

### 6.3. Constraint of the Lifetime for ESS

A lifetime of the residential ESS is an important constraint for the HEMS problem, and it is expressed as the SOE range in terms of the number of the limited charging and discharging cycles of ESS [39]. A key part of this task would be to identify the proper limit of charging and discharging cycles of ESS. To this end, one possible direction in the proposed framework is to add the limit of charging and discharging cycles to dissatisfaction cost for the ESS agent. This enables the ESS agent to determine the policy that maintains the number of charging and discharging cycles within its acceptable range.

## 7. Conclusions

We have proposed a machine learning-based smart home energy management algorithm using reinforcement learning and an artificial neural network. The proposed algorithm can minimize the electricity bill through the energy consumption scheduling of two controllable home appliances (an air conditioner and a washing machine) and the charging and discharging of the energy storage system, while maintaining the consumer comfort level and appliance operation characteristics. In the proposed Q-learning framework, the agents for a washing machine, an air conditioner, and an energy storage system independently learn their actions through the interaction of an environment until they maximize the total cumulative rewards received from the environment. The washing machine agent schedules the energy consumption of the washing machine within the consumer preferred operation period. The energy storage system agent calculates the charging and discharging energy while preventing the overcharging and undercharging of the energy storage system. In the indoor temperature prediction model constructed by an artificial neural network, the air conditioner agent performs the scheduling for the energy consumption of the air conditioner while satisfying the consumer preferred indoor temperature. The performance of the proposed algorithm was validated in the simulation study, and the results confirm the economical advantages of the proposed approach compared to the existing optimization approach using mixed-integer linear programming.

In future work, we plan to develop a multi-agent reinforcement learning algorithm that schedules the energy consumption of multiple smart homes with distributed energy resources and smart home appliances. A key challenge lies in how to design the efficient communication scheme between multiple smart homes for achieving the energy savings and maintaining the consumer comfort level. In addition, the practical implementation of the developed algorithm should be tested in large-scale realistic electric power networks. Last but not least, we plan to integrate advanced neural network models such as recurrent neural networks and long short-term memory in the proposed framework to improve the prediction accuracy of the indoor temperature.

## Nomenclature

The main notations are summarized below. Other undefined symbols are explained in the text:

$\pi_{t}$	TOU price at time slot *t*
ϵ	Penalty for consumer thermal discomfort cost
$E_{a,t}$	Energy consumption of appliance *a* at time slot *t*
$E_{t}^{\mathrm{net}}$	Net energy consumption at time slot *t*
$E_{a}^{\max(\min)}$	Maximum (Minimum) energy consumption of appliance *a*
$E_{a,t}^{\mathrm{ch}}$	Charging energy of ESS *a* at time slot *t*
$E_{a,t}^{\mathrm{dch}}$	Discharging energy of ESS *a* at time slot *t*
$E_{a}^{\mathrm{ch},\max(\min)}$	Maximum (Minimum) charging energy of ESS *a*
$E_{a}^{\mathrm{dch},\max(\min)}$	Maximum (Minimum) discharging energy of ESS *a*
$SOE_{a,t}$	State of energy of ESS *a* at time slot *t*
$SOE_{a}^{\max(\min)}$	Maximum (Minimum) state of energy of ESS *a*
$\eta_{a}^{\mathrm{ch}}$	Charging efficiency of ESS *a*
$\eta_{a}^{\mathrm{dch}}$	Discharging efficiency of ESS *a*
${\hat{E}}_{t}^{\mathrm{PV}}$	Predicted PV generation energy at time slot *t*
$T_{t}^{\mathrm{in}}$	Indoor temperature at time slot *t*
${\hat{T}}_{t}^{\mathrm{out}}$	Predicted outdoor temperature at time slot *t*
$T^{\max(\min)}$	Maximum (Minimum) preferred indoor temperature
α,β	Thermal characteristic for AC
$T^{\mathrm{set}}$	Consumer preferred indoor temperature
$\omega_{s}^{\mathrm{pref}}$	Consumer preferred starting time for WM
$\omega_{f}^{\mathrm{pref}}$	Consumer preferred finishing time for WM
$b_{a,t}^{c,I}$	Binary charging and discharging state of ESS *a* at time slot *t*: “1” for charging, “0” for discharging
$b_{a,t}^{c,NI}$	Binary consumption state of non-interruptible shiftable appliance *a* at time slot *t*:
	“1” for consumption, “0” otherwise
$s_{t}$	State at time slot *t*
$a_{t}$	Action at time slot *t*
$r_{t}^{\mathrm{WM}}$	Reward of WM agent at time slot *t*
$r_{t}^{\mathrm{AC}}$	Reward of AC agent at time slot *t*
$r_{t}^{\mathrm{ESS}}$	Reward of ESS agent at time slot *t*
θ	Learning rate in RL
γ	Discount factor in RL
$\Delta E^{\mathrm{AC}}$	Energy unit in discrete set of actions for AC agent
$\Delta E^{\mathrm{ESS}}$	Energy unit in discrete set of actions for ESS
$\bar{\delta }\left(\underline{\delta }\right)$	Penalty for early (late) operation of WM in reward function
$\bar{\tau }\left(\underline{\tau }\right)$	Penalty for overcharging (undercharging) of ESS in reward function
κ	Penalty for consumer thermal discomfort cost in reward function
T	Set of time slots
A	Set of appliances
$A_{r}^{c}$	Set of reducible appliances
$A_{s}^{c,NI}$	Set of shiftable appliances with non-interruptible loads
$A_{s}^{c,I}$	Set of shiftable appliances with interruptible loads
$A^{uc}$	Set of uncontrollable appliances
$S^{WM}$	Set of states for WM
$S^{AC}$	Set of states for AC
$S^{ESS}$	Set of states for ESS
$A^{WM}$	Set of actions for WM
$A^{AC}$	Set of actions for AC
$A^{ESS}$	Set of actions for ESS
